# A new subfamily LIP of the major intrinsic proteins

**DOI:** 10.1186/1471-2164-15-173

**Published:** 2014-03-04

**Authors:** Kirill Vladimirovich Khabudaev, Darya Petrovna Petrova, Mikhail Aleksandrovich Grachev, Yelena Valentinovna Likhoshway

**Affiliations:** Department of Cell Ultrastructure, Limnological Institute, Siberian Branch of the Russian Academy of Sciences, 3 Ulan-Batorskaya, P.O. Box 278, 664033 Irkutsk, Russia

**Keywords:** Aquaporins, Chromista, Heterokontophyta, Major intrinsic proteins

## Abstract

**Background:**

Proteins of the major intrinsic protein (MIP) family, or aquaporins, have been detected in almost all organisms. These proteins are important in cells and organisms because they allow for passive transmembrane transport of water and other small, uncharged polar molecules.

**Results:**

We compared the predicted amino acid sequences of 20 MIPs from several algae species of the phylum Heterokontophyta (Kingdom Chromista) with the sequences of MIPs from other organisms. Multiple sequence alignments revealed motifs that were homologous to functionally important NPA motifs and the so-called ar/R-selective filter of glyceroporins and aquaporins. The MIP sequences of the studied chromists fell into several clusters that belonged to different groups of MIPs from a wide variety of organisms from different Kingdoms. Two of these proteins belong to Plasma membrane intrinsic proteins (PIPs), four of them belong to GlpF-like intrinsic proteins (GIPs), and one of them belongs to a specific MIPE subfamily from green algae. Three proteins belong to the unclassified MIPs, two of which are of bacterial origin. Eight of the studied MIPs contain an NPM-motif in place of the second conserved NPA-motif typical of the majority of MIPs. The MIPs of heterokonts within all detected clusters can differ from other MIPs in the same cluster regarding the structure of the ar/R-selective filter and other generally conserved motifs.

**Conclusions:**

We proposed placing nine MIPs from heterokonts into a new group, which we have named the LIPs (large intrinsic proteins). The possible substrate specificities of the studied MIPs are discussed.

**Electronic supplementary material:**

The online version of this article (doi:10.1186/1471-2164-15-173) contains supplementary material, which is available to authorized users.

## Background

The major intrinsic proteins (MIPs) [1], or aquaporins [2], allow for the passive transmembrane transport of water and other small, uncharged polar molecules [3]. Glyceroporin (GlpF) from *Escherichia coli*[4] and aquaporin 1 (AQP1) from bovine [5] were the first MIPs for which the 3D structures were established through X-ray crystallographic analysis. The similarities between proteins of the MIP family suggest that they have a common origin [6]. Plant aquaporins comprise a large protein family [7–9]. The topology of MIPs resembles a sandwich, consisting of six transmembrane α-helical strands (denoted H1 through H6). These strands are connected to each other by five loops (denoted LA through LE). The LB and LE loops each consist of a short α-helix connected by highly conserved NPA-motifs, and these loops are partly located within the membrane [10]. Certain amino acid residues (a.a.) of the H2 and H5 strands, together with two a.a. of the LE site in the same plane, form the so-called ar/R-filter (aromatic/arginine), which determines the substrate specificity of the protein [4, 5, 11]. Certain sub-families of MIPs contain conserved a.a. within the ar/R-filter [12, 13]: e.g. F56, H180, C189, and R195 in the aquaporin HsAQP1 [5]; and W48, G191, F200, and R205 in the glyceroporin EcGlpF [4]. The pore diameter of the latter is larger than the former [14]. It was recently shown that substitution of a.a. within the ar/R-filter results in a change in substrate specificity or in a loss of function [15]. However, the design of the ar/R filter is not the only determinant of specificity. Determinants other than the ar/R filter before experimental studies cannot be identified by theoretical analysis for a majority of MIPs which are considered in the present study.

Diatoms are unicellular, phototrophic, eukaryotic organisms that are present in all marine and freshwater habitats. They originated as a result of double endosymbiosis followed by long-term (240 million years) evolution [16–18], which resulted in the migration of many genes, such as bacterial genes, into the diatom nuclear genome [19, 20]. A general feature of diatoms is the presence of an intricately ornamented cell wall, known as a frustule, which consists of silica. The synthesis of the solid and nearly anhydrous elements of the frustule takes place within specialised sub-cellular vesicles (silica deposition vesicules, SDVs) [21, 22]. Maturation of the frustule requires the removal of water from the SDV. It has been proposed that this process is mediated by aquaporins [23].

In this study we investigate MIPs from Chromista, phylum (Infrakindom) Heterokontophyta (Additional file 1). It was proposed that all pigmented heterokonts appeared due to double symbiosis and the simultaneous appearance of the ability to build cell walls with silica [24]. We used taxonomy from algae base [25].

Ten MIP genes were found in the complete genome sequences for the diatoms *Thalassiosira pseudonana* TpMIP1, TpMIP2 [19] and *T. oceanica* ToMIP1, ToMIP2 [26] (class Coscinodiscophyceae), *Phaeodactylum tricornutum* PtMIP1, PtMIP2, PtMIP3, PtMIP4, PtMIP5 [20] (class Bacillariophyceae), and *Nannochloropsis gaditana* NgMIP [27] (class Eustigmatophyceae). A MIP gene was also recently discovered in the genome of the freshwater araphid pennate diatom *Synedra acus* subsp. *radians* (class Fragillaryophyceae). The length of the predicted SarMIP is 286 a.a. [28]. MIP genes were found in the genomes of the diatoms *Pseudo-nitzschia multiseries*[29] and *Fragilariopsis cylindrus*[30] (class Bacillariophyceae).

The database dedicated to Major Intrinsic Proteins MIPdb [31] contains seven MIP sequences from *Ectocarpus siliculosus*, EsAQP, EsPIP, and EsMIP (class Phaeophyceae), as well as from *Aureococcus anophagefferens*, AaMIP1, AaMIP2, AaMIP3, and AaMIP4 (class Pelagophyceae).

The purpose of the present study was to compare the predicted a.a. sequences of 20 MIPs from these algae with MIP sequences from a wide variety of organisms. We show that MIPs from heterokonts belong to different subfamilies, and nine of them merge into a new Large intrinsic protein (LIP) subfamily, which is closely related with the SIP subfamily [32] and the MIPC subfamily [33].

## Results

### Search for homologues in the MIPdb

We used the MIPdb to find homologues of the 20 MIPs from heterokonts. This database contains 8429 MIPs belonging to 11 groups (subfamilies): 577 AQPes, 1150 AQPps, 363 GLAes, 1827 GLAps, 1052 GLPps, 192 NIPs, 661 PIPs, 42 SIPs, and 375 TIPs, as well as 1053 predicted MIPs. There are 1137 sequences that are unclassified MIPs. MIPdb contains 16 MIPs from heterokonts, 15 of them belonging to the unclassified group and one of them (EsPIP) belonging to AQPe (Additional file 1).

To find closely related sequences, we used the phmmer procedure in the HMMER3-package. We selected the proteins with the smallest e-values from each of the MIPdb groups. These proteins are hypothesised to be related to the 20 MIPs studied.

### Phylogenetic analysis

To classify the MIPs from heterokonts, their a.a. sequences were subjected to a phylogenetic analysis together with proteins selected based on the results of a search for homologues and reference sequences, which included a typical aquaporin (HsAQP1), a glyceroporin (EcGlpF), and NIPs from rice and maize (OsNIP21, OsNIP22, ZmNIP22, and ZmNIP23). It has been showed that these four NIPs are transporters of silicic acid [34, 35]. Among the 22 analysed MIPs from green algae, we identified seven different groups, including PIPs, GlpF-like intrinsic proteins, and MIPs unique to green algae (MIPA to MIPE) [33]. These proteins have also been analysed in order to compare the results. The total number of sequences subjected to phylogenetic analysis was 212. Alignment was performed using the profile of the MIP PF00230 in the Pfam database.

**Figure 1:**
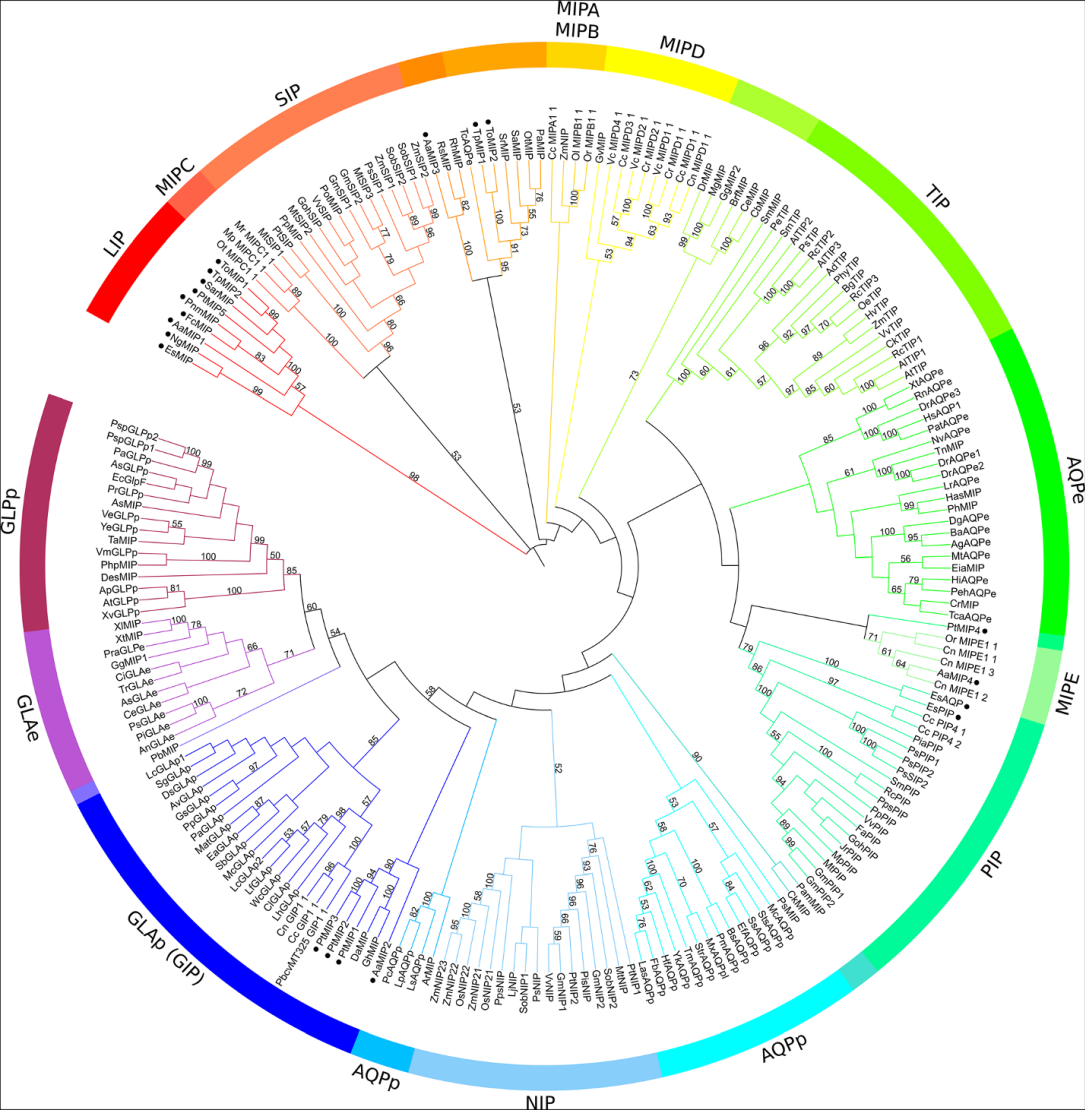
**Phylogenetic tree of 212 MIP sequences constructed using the neighbour joining (NJ) method and the Jones-Taylor-Thornton (JTT) evolutionary model (• - heterokont proteins).**

The results of the phylogenetic analysis (Figure 1) demonstrated that the topology of the tree constructed from the selected sequences corresponds to the modern understanding of the phylogeny of MIPs [36]. MIPs from heterokonts fall into different clades.

Three sequences (PtMIP1, PtMIP2, and PtMIP3) from *P. tricornutum*, and AaMIP2 from the *A. anophagefferens* clustered within a large clade that includes GLAp, GLAe, and GLPp with a bootstrap support of 58%.

The sequences of TpMIP1 from *T. pseudonana*, ToMIP2 from *T. oceanic*, and AaMIP3 from *A. anophagefferens* clustered with unclassified MIPs from bacteria with bootstrap supports of 52% and 100%, respectively.

Two sequences (EsAQP and EsPIP) from the brown alga *E. siliculosus* clustered with plant PIPs with a bootstrap support of 79%. Nine sequences (EsMIP, NgMIP, AaMIP1, PtMIP5, PnmMIP, FcMIP, SarMIP, TpMIP2, and ToMIP1) constituted a separate clade with a bootstrap support of 98%. Their closest relatives are the plant SIPs and MIPC from green algae. AaMIP4 from *A. anophagefferens* clustered with MIPE from green algae with a bootstrap support of 71%. Only one sequence of PtMIP4 did not reliably belong to any clade. EsAQP and EsPIP clustered with CcPIP4;1 and CcPIP4;2 from green algae. AaMIP2, PtMIP1, PtMIP2, and PtMIP3 clustered with GIPs from green algae as a sister clade with a bootstrap support of 58%.

### Peculiarities of the structures of MIPs from heterokonts

**Figure 2:**
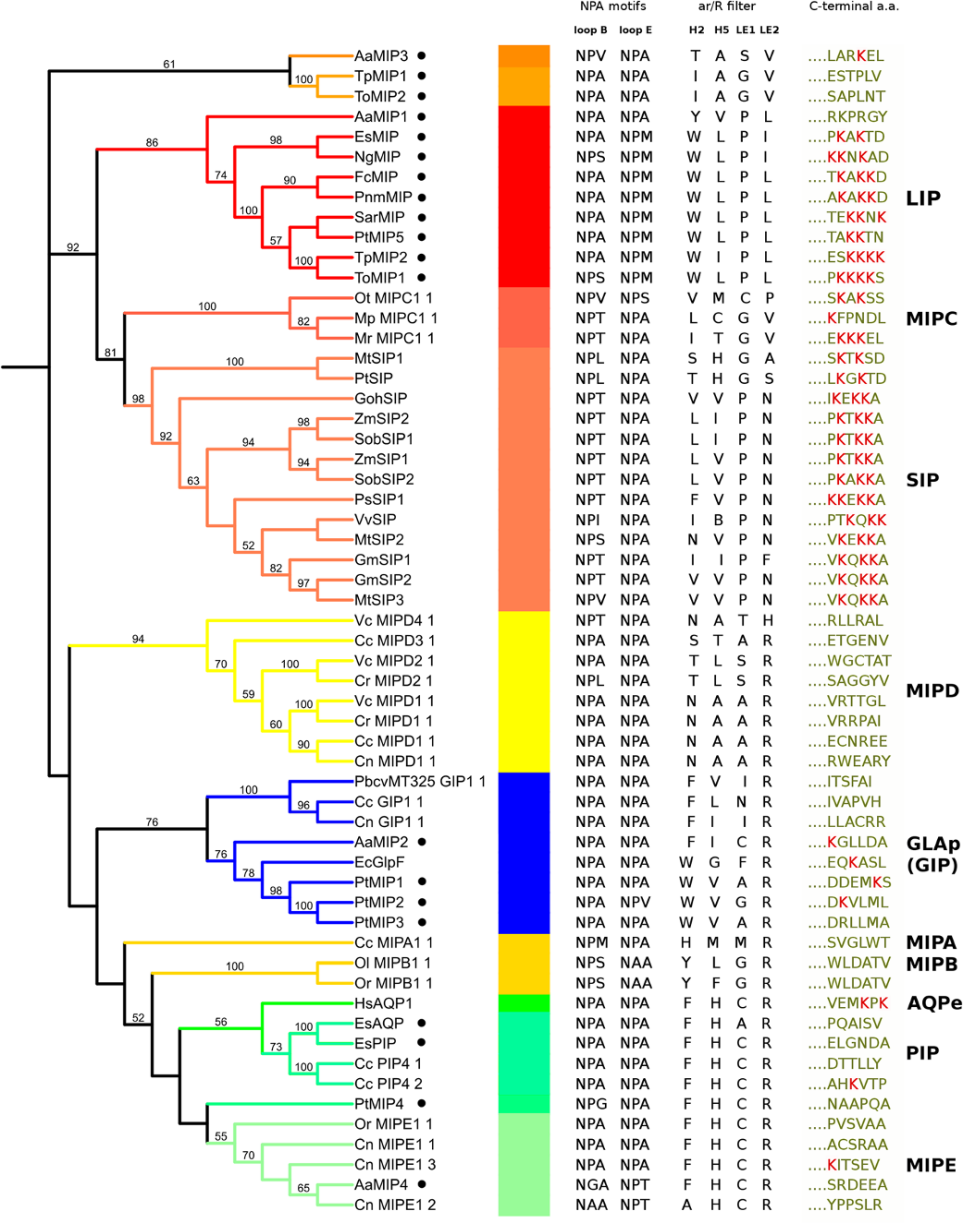
**Comparison of NPA motifs, ar/R filters, and C-terminal a.a. of 20 MIPs from heterokonts with some MIPs of different subfamilies.** A phylogenetic tree was constructed using the neighbour joining (NJ) method and the Jones-Taylor-Thornton (JTT) evolutionary model (• - heterokont proteins; lysine residues are marked in red).

Of the 20 heterokonts’ MIPs studied (Figure 2), only eight contain a pair of conserved NPA motifs. It worth noting that the EsMIP, NgMIP, PtMIP5, PnmMIP, FcMIP, SarMIP, TpMIP2, and ToMIP1 proteins, which are the closest to SIPs, all have an NPM motif in place of the second NPA. However, the second motif of SIPs is an NPA and the first motif is variable in the third a.a. position. AaMIP4 has NGA instead the first NPA motif. The MIPC sequences contain the first modified motif NP[T/V].

The amino acids belonging to the ar/R filters are shown in Additional file 1 and Table 1 and Figure 2. MIPs 1, 2, and 3 from *P. tricornutum* have a.a. compositions in the ar/R filter that are similar to the GLAp MIP, whereas the ar/R filter of AaMIP2, which was in the same clade as GLAp, GLAe, and GLPp, has the same a.a. composition as human aquaporin HsAQP1 at positions H2, LE1, and LE2.

**Table 1:** **Selectivity of the MIPs of heterokonts based on the similarity of their ar/R filter to the ar/R filters of the MIPs with known selectivity**

Subfamily	Protein	Ar/R selectivity filter				Substrate specificity
		H2	H5	LE1	LE2	
GLAp (GIP)	AaMIP2	F	I	C	R	Unknown
	PtMIP1	W	V	A	R	Glycerol permease [36]
	PtMIP2	W	V	G	R	
	PtMIP3	W	V	A	R	
PIP	EsAQP	F	H	A	R	Water [5]
	EsPIP	F	H	C	R	
MIPE	AaMIP4	F	H	C	R	Water [5]
LIP	AaMIP1	Y	V	P	L	Unknown
	EsMIP	W	L	P	I	
	NgMIP	W	L	P	I	
	FcMIP	W	L	P	L	
	PnmMIP	W	L	P	L	
	SarMIP	W	L	P	L	
	PtMIP5	W	L	P	L	
	TpMIP2	W	I	P	L	
	ToMIP1	W	L	P	L	
Unknown	PtMIP4	F	H	C	R	Water [5]
	AaMIP3	T	A	S	V	Unknown
	TpMIP1	I	A	G	V	
	ToMIP2	I	A	G	V	

The amino acid composition of the ar/R filters of PtMIP4, AaMIP4, and EsPIP was identical to that of the ar/R filter of human aquaporin HsAQP1. The ar/R filter of EsAQP differs from the ar/R filter of PtMIP4, AaMIP4, and EsPIP by the presence of an alanine (A) in place of the cysteine (C) at position LE1. The LE1 position in the filters of EsMIP, NgMIP, PtMIP5, PnmMIP, FcMIP, AaMIP1, SarMIP, TpMIP2, and ToMIP1, as well as a majority of the filters of the sister clade, was occupied by a proline (P). Unlike the other MIPs, their LE2 positions are occupied by leucine (L) or isoleucine (I). Hence, EsMIP, NgMIP, PtMIP5, PnmMIP, FcMIP, AaMIP1, SarMIP, TpMIP2, and ToMIP1 differed from the other MIPs in this feature. The H2 strand of the filter includes tryptophan (W)/tyrosine (Y) in these nine proteins, which is a more typical feature of GLPp, GLAp, and NIPs that are not able to transport silicic acid (Additional file 1).

## Discussion

Phylogenetic analysis of MIP a.a. sequences predicted from the nucleotide sequences of the respective genes, as well as a comparison of the ar/R filters of 20 MIPs from heterokonts, revealed that some of these proteins have very close homologues among the 8429 proteins documented in the MIPdb. On the basis of the ar/R filter, substrate specificity could be suggested.

Three proteins (PtMIP1, PtMIP2, and PtMIP3) from the diatom *P. tricornutum* were classified as GlpF-like intrinsic proteins. Their closest homologues are DaMIP, which is from the sulphate reducing anaerobic proteobacteria *Desulfuromonas acetoxidans*, and GhMIP, which is from the Gram-positive coccus *Gemella haemolysans*. The ar/R filters of PtMIP1, PtMIP2, and PtMIP3 are identical to those of NIPs of subgroup I, which have glycerol permease activity (Table 1) [12, 37].

AaMIP2 from *A. anophagefferens* also belongs to the GlpF-like intrinsic proteins, as revealed by the phylogenetic analysis (Figure 1). However, three a.a. in the ar/R filter in the H2, LE1, and LE2 of AaMIP2 are identical to those found in the human aquaporin HsAQP1.

Two proteins, EsAQP and EsPIP, of the *E.* s*iliculosus* belong to the PIP subfamily, as revealed by the phylogenetic analysis (Figure 1). Unlike EsAQP and EsPIP, all other PIPs are highly conserved [38, 33]. The composition of the ar/R filter of EsAQP and EsPIP is different from that of the ar/R filter of PIPs at some positions (Additional file 1). However, the composition of the ar/R filters of EsPIP and HsAQP1 is identical. We propose that, based on their sequence composition, these two proteins from brown alga *E.* s*iliculosus* are intermediate forms between the human HsAQP1 and plant PIPs.

AaMIP4 from *A. anophagefferens* clusters with a specific subfamily MIPE from green algae on the phylogenetic tree. The ar/R filters of AaMIP4 and MIPEs are identical to those of HsAQP1. This similarity suggests these proteins have specificities for water.

A phylogenetic analysis revealed that PtMIP4 from the diatom *P. tricornutum* does not have close relatives. The sequence of this protein differs from classical aquaporins in that the first NPA motif in PtMIP4 is transformed into NPG, but all residues of the ar/R filter are identical to those of the HsAQP1 and MIPEs. TpMIP1 and ToMIP2 from diatoms and AaMIP3 from *A. anophagefferens* cluster with bacterial MIPs on the phylogenetic tree. Multiple alignments have shown that the ar/R filters of bacterial MIPs, TpMIP1, ToMIP2, and AaMIP3 differ from those found in the proteins from other subfamilies (Additional file 1). The functions of these bacterial MIPs are not yet known. Therefore, no function can be proposed at this time for TpMIP1, ToMIP2, or AaMIP3. Interestingly, the aquaporin TcAQPe of the parasitic trypanosome *Trypanosoma cruzi* falls into the same clade, although its ar/R filter [14] is different from that of typical aquaporins at all four positions.

Nine proteins (EsMIP, NgMIP, PtMIP5, PnmMIP, FcMIP, AaMIP1, SarMIP, TpMIP2, and ToMIP1) form a separate clade adjacent to the SIP clade of plants and the MIPC clade of green algae. Ishibashi et al. [39] concluded that during their evolution, SIPs and XIPs lost conservation of the NPA motifs. The first motif that replaced NPA in SIPs was NP[T/L/S/I], while in MIPC it was NP[T/V]. The motif that is found in place of the second NPA in these nine proteins is NPM. We found four other proteins with NPM in place of the second NPA in the MIPdb. One of these proteins belongs to the NIP subfamily, and the other three belong to an uncharacterised group. However, arginine (R) is C–terminal to NPM in the LE2 position in these four proteins, whereas leucine (L) or isoleucine (I) are C-terminal to NPM in MIPs from heterokonts. The LE1 position in the ar/R filters of EsMIP, NgMIP, PtMIP5, PnmMIP, FcMIP, AaMIP1, SarMIP, TpMIP2, and ToMIP1 are occupied by the same a.a. as those in these positions in SIPs. However, the sites at positions H2 and LE2 are occupied by different a.a. from those found in the same positions in the SIPs. Remarkably, a tryptophan (W) at position H2 occurs in all glyceroporins, as well as in NIPs that are not able to transport silicic acid (Additional file 1). Residues of the ar/R filter of MIPC do not match any one a.a. position of the ar/R filter of these nine proteins. Similarities were revealed in the terminal a.a. of lysine (K) in SIPs and EsMIP, NgMIP, PtMIP5, PnmMIP, FcMIP, SarMIP, TpMIP2, and ToMIP1 (Figure 2). Of all the MIPCs, only one sequence (MrMIPC1;1) contains a terminal lysine (K).

On the basis of the above evidence, we suggest a new phylogenetic clade, LIPs, which includes nine proteins: EsMIP, NgMIP, PtMIP5, PnmMIP, FcMIP, AaMIP1, SarMIP, TpMIP2, and ToMIP1 (Figure 1). This new clade has high bootstrap support. Indeed, seven of the nine proteins of the subfamily are large (280 to 317 a.a.), with EsMIP consisting of 225 a.a. and NgMIP consisting of 230 a.a. (Additional file 1). However, EsMIP and NgMIP are similar to other LIPs in terms of phylogeny (Figure 1) and the structure of the ar/R filters (Figure 2).

Heterokonts are thought to be derived from a secondary endosymbiotic process between a red alga and a heterotrophic eukaryote [19]. Recent studies of the genomes of the diatoms have revealed the participation of green algae in the origin of some membrane transporters [40]. We showed that of the MIPs from heterokonts, one protein (AaMIP4) has a relationship with a specific subfamily MIPE from green algae.

According to our analysis, none of the 20 analysed MIPs from heterokonts are relatives to NIPs, which transports silicic acid, on the phylogeny (Figure 1), and have dissimilar ar/R filter (Additional file 1 and Table 1).

## Conclusions

Heterokonts, like other organisms, contain a variety of MIPs, which could allow for the transport of substances, such as water, glycerol, urea, carbon dioxide, etc. We found that heterokonts contain MIPs that belong to different subfamilies, such as PIP, GIP, and MIPE. The most surprising finding is that during their evolution, heterokonts acquired unique genes, such as those that encode the MIPs of the LIP subfamily. These unusual proteins encoded by these genes are only distantly related to typical aqua- or glyceroporins, and are characterised by a specific motif and the composition of the ar/R filter. Notably, none of MIPs from heterokonts have any similarities with NIPs that are responsible for transporting silicic acid.

## Methods

### Search for homology

A search for closely related MIPs was carried out with MIPdb, which is a motif-oriented database that allows for analyses on the biological, structural, and functional levels and is used to identify highly specific domains of unknown proteins. To analyse the similarities between MIPs from heterokonts and MIP sequences from a wide variety of organisms, we used HMMER3 [41] with the procedure phhmer to carry out a BLAST-like search for a specified sequence in the database.

### Alignments and phylogenetic analysis

Multiple sequence alignments of aquaporin amino acids on the Pfam profile of the MIP family PF00230 was carried out using HMMER3 with the procedure hmmalign. The resulting alignment was edited in the JalView program [42] to remove non-informative C- and N-termini. The phylogenetic trees were constructed using MEGA5 v5.1 [43] using the bootstrap neighbour-joining (NJ) method with 1000 replicates and the Jones-Taylor-Thornton (JTT) model. For tree visualisation, we used iTOL [44]. Multiple sequence alignments of the MIPs of heterokonts were carried out using the program Muscle v3.8.31 [45].

### Availability of supporting data

The data sets supporting the results of this article are available in the DRYAD repository, http://dx.doi.org/10.5061/dryad.8f61v.

## Electronic supplementary material

Additional file 1: **Names of the organisms, short titles of MIPs, identificator from MIPdb and the structural characteristics of amino acid sequences (• - proteins of heterokonts).** (DOCX 44 KB)

## Abbreviations

AQP: Aquaporin
AQPe: Aquaporin of eukaryotes
AQPp: Aquaporin of prokaryotes
GIP: GlpF-like intrinsic protein
GLAe: Aquaglyceroporin of eukaryotes
GLAp: Aquaglyceroporin of prokaryotes
GLPp: Glycerol uptake facilitator of prokaryotes
GlpF: Glycerol uptake facilitator protein
MIP: Major intrinsic protein
LIP: Large intrinsic protein
MIPdb: MIP data base
NIP: Nodulin 26-like intrinsic protein
PIP: Plasma membrane intrinsic protein
SIP: Small basic intrinsic protein
SDV: Silica deposition vesicle
TIP: Tonoplast intrinsic protein
XIP: X intrinsic protein.
