# Supplementary material for: A new subfamily LIP of the major intrinsic proteins
Source: BMC Genomics. 2014 Mar 4;15(1):173. doi: 10.1186/1471-2164-15-173 (PMC4022174; doi:10.1186/1471-2164-15-173)
Supplement: Supplementary file 1 — Additional file 1: Names of the organisms, short titles of MIPs, identificator from MIPdb and the structural characteristics of amino acid sequences (• - proteins of heterokonts). (DOCX 44 KB) [file 12864_2013_7019_MOESM1_ESM.docx]

Additional file 1: Table S1. Names of the organisms, short titles of MIPs, identificator from MIPdb and the structural characteristics of amino acid sequences (• - proteins of heterokonts).

| Organism | Abbreviation | ID MIPdb (* - accession number of GenBank) | Length, a.a. | Group of MIPdb | First/second NPA motifs | A.a. of the ar/R filter | | | |
| --- | --- | --- | --- | --- | --- | --- | --- | --- | --- |
|  |  |  |  |  |  | H2 | H5 | LE1 | LE2 |
| Acetobacter pasteurianus subsp pasteurianus | ApGLPp | H1UR08_ACEPA | 272 | GLPp | NPA/NPA | W52 | G194 | W203 | R209 |
| Acetobacter tropicalis | AtGLPp | F7VJ95_9PROT | 263 | GLPp | NPA/NPA | W41 | G183 | W192 | R198 |
| Acinetobacter sp NBRC 100985 | AsMIP | G7GAU1_9GAMM | 280 | predicted | NPA/NPA | W49 | G192 | F201 | R207 |
| Aerococcus viridans | AvGLAp | D4YGZ8_9LACT | 234 | GLAp | NPA/NPA | W44 | G173 | Y182 | R188 |
| Aggregatibacter segnis | AsGLPp | E6KXD4_9PAST | 264 | GLPp | NPA/NPA | W48 | G189 | F198 | R204 |
| Anopheles gambiae | AgAQPe | D1MYR4_ANOGA | 259 | AQPe | NPA/NPA | F53 | H177 | S186 | R192 |
| Arabidopsis lyrata subsp lyrata | AlTIP1 | D7KFJ5_ARALY | 267 | TIP | NPA/NPA | H73 | T195 | A204 | R210 |
| Arabidopsis lyrata subsp lyrata | AlTIP2 | D7KIP4_ARALY | 248 | TIP | NPA/NPA |  | I178 | G187 | R193 |
| Arabidopsis thaliana | AtTIP | TIP32_ARATH | 267 | TIP | NPA/NPA | H73 | I195 | A204 | R210 |
| Arabidopsis_lyrata | AlTIP3 | D7LBI2_ARALY | 249 | TIP | NPA/NPA | H60 | I181 | A190 | R196 |
| Arachis diogoi | AdTIP | E3NYH3_9FABA | 177 | TIP | NPA/NPA | - | I113 | A122 | V128 |
| Ascaris suum | AsGLAe | F1L4B4_ASCSU | 336 | GLAe | NPA/NPA | W59 | G198 | Y207 | R213 |
| Aspergillus niger | AnGLAe | A2QF45_ASPNC | 285 | GLAe | NPA/NPA | W53 | G199 | Y208 | R214 |
| •Aureococcus anophagefferens | AaMIP1 | F0Y1P0_AURAN | 317 | putative | NPA/NPA | Y63 | V188 | P197 | L203 |
| •Aureococcus anophagefferens | AaMIP2 | F0Y4Q5_9STRA | 266 | putative | NPA/NPA | F55 | I202 | C211 | R217 |
| •Aureococcus anophagefferens | AaMIP3 | F0YHU9_AURAN | 250 | putative | NPV/NPA | T66 | A178 | S187 | V193 |
| •Aureococcus anophagefferens | AaMIP4 | F0YEV7_AURAN | 249 | putative | NGA/NPT | F43 | H169 | C178 | R184 |
| Bacteroides sp 1_1_6 | BsAQPp | C6IQS8_9BACE | 222 | AQPp | NPA/NPA | F38 | H163 | T172 | R178 |
| Belgica antarctica | BaAQPe | F5HRA1_9DIPT | 291 | AQPe | NPA/NPA | F85 | H212 | S221 | R227 |
| Branchiostoma_floridae | BrfMIP | C3YWG4_BRAFL | 221 | unclassified | NPA/NPA | H47 | I173 | A182 | R188 |
| Bruguiera gymnorhiza | BgTIP | B1Q4T7_9ROSI | 212 | TIP | NPA/NPA | H24 | I146 | A155 | V161 |
| Caenorhabditis elegans | CeGLAe | Q7JMQ6_CAEEL | 285 | GLAe | NPA/NPA | F58 | G197 | Y206 | R212 |
| Caenorhabditis_brenneri | CbMIP | G0N8R0_CAEBE | 245 | putative | NPA/NPA | H49 | I173 | A182 | R188 |
| Caenorhabditis_elegans | CeMIP | G5EEK0_CAEEL | 273 | putative | NPA/NPA | H81 | I205 | A214 | R220 |
| Caligus rogercresseyi | CrMIP | C1BNY4_9MAXI | 255 | unclassified | NPA/NPA | F60 | H186 | S195 | R201 |
| Candidatus Kuenenia | CkMIP | Q1PX58_9BACT | 247 | putative | NPA/NPA | Y48 | V172 | G181 | R187 |
| Ciona intestinalis | CiGLAe | F6SXP0_CIOIN | 310 | GLAe | NPA/NPA | Y67 | G207 | Y216 | R222 |
| Clostridium ljungdahlii | ClGLAp | D8GTH2_CLOLD | 242 | GLAp | NPA/NPA | Y49 | V181 | P190 | R196 |
| Cucurbita cv Kurokawa Amakuri | CkTIP | Q39647_9ROSI | 269 | TIP | NPA/NPA | H77 | I199 | A208 | R214 |
| Danio rerio | DrAQPe2 | Q6AZD2_DANRE | 320 | AQPe | NPA/NPA | F79 | H203 | A212 | R218 |
| Danio rerio | DrAQPe3 | F1QQB8_DANRE | 269 | AQPe | NPA/NPA | F51 | H174 | C183 | R189 |
| Danio rerio | DrAQPe1 | Q5SNS8_DANRE | 308 | AQPe | NPA/NPA | F67 | H191 | A200 | R206 |
| Danio_rerio | DrMIP | A7MCF8_DANRE | 260 | unclassified | NPA/NPA | H73 | I199 | A208 | R214 |
| Desmospora_sp | DesMIP | F5SCX1_9BACL | 273 | unclassified | NPA/NPA | W46 | G191 | F200 | R206 |
| Desulfovibrio salexigens | DsGLAp | C6C1M8_DESAD | 235 | GLAp | NPA/NPA | W44 | G173 | Y182 | R188 |
| Desulfuromonas acetoxidans | DaMIP | Q1K2J6_DESAC | 271 | unclassified | NPA/NPA | W44 | I193 | A202 | R208 |
| Drosophila grimshawi | DgAQPe | B4J8Z8_DROGR | 264 | AQPe | NPA/NPA | F55 | H179 | A188 | R194 |
| •Ectocarpus siliculosus | EsAQP | D8LTV5_ECTSI | 278 | unclassified | NPA/NPA | F84 | H209 | A218 | R224 |
| •Ectocarpus siliculosus | EsPIP | D8LTV4_ECTSI | 280 | AQPe | NPA/NPA | F109 | H204 | C213 | R219 |
| •Ectocarpus siliculosus | EsMIP | D7G8A1_ECTSI | 225 | putative | NPA/NPM | W38 | I159 | P171 | I177 |
| Eisenia andrei | EiaMIP | C0MP57_9ANNE | 292 | putative | NPG/NPA | F71 | H195 | S204 | R210 |
| Elizabethkingia anophelis | EaGLAp | H0KQC3_9FLAO | 243 | GLAp | NPA/NPA | W44 | G184 | Y193 | R199 |
| Enterococcus faecalis | EfAQPp | E0G1L8_ENTFA | 216 | AQPp | NPA/NPA | F35 | H159 | T168 | R174 |
| Escherichia coli | EcGLPp | GLPF_ECOLI | 281 | GLPp | NPA/NPA | W48 | G191 | F200 | R206 |
| Flavobacteriales bacterium | FbAQPp | A4ATY3_9FLAO | 229 | AQPp | NPA/NPA | F42 | H171 | T180 | R186 |
| Fragaria ananassa | FaPIP | D8V5R9_FRAAN | 285 | PIP | NPA/NPA | F87 | H216 | T225 | R231 |
| •Fragilariopsis cylindrus | FcMIP | - | 285 | - | NPA/NPM | W76 | L200 | P209 | L215 |
| Gallus gallus | GgMIP1 | F1NZ32_CHICK | 229 | unclassified | NPA/NPA |  | T143 | C152 | R158 |
| Gallus gallus | GgMIP2 | F1NA56_CHICK | 254 | unclassified | NPV/NPA | H67 | I193 | P202 | R208 |
| Gardnerella vaginalis | GvMIP | D2RB62_GARV4 | 323 | putative | NPA/NPA | T58 | A212 | A221 | R227 |
| Gemella haemolysans | GhMIP | F3A2Y4_9BACL | 270 | unclassified | NPA/NPT | W47 | L195 | A204 | R210 |
| Geobacillus_sp | GsGLAp | D7CYX7_9BACI | 272 | GLAp | NPA/NPA | W44 | G173 | Y182 | R188 |
| Glycine max | GmNIP1 | C6TL07_SOYBN | 273 | NIP | NPA/NPA | W80 | V200 | A209 | R215 |
| Glycine max | GmPIP1 | C6TI29_SOYBN | 287 | PIP | NPA/NPA | F89 | H218 | T227 | R233 |
| Glycine max | GmPIP2 | C6TLJ6_SOYBN | 287 | PIP | NPA/NPA | F89 | H218 | T227 | R233 |
| Glycine max | GmSIP1 | C6TJ95_SOYBN | 247 | SIP | NPT/NPA | I58 | I181 | P190 | F196 |
| Glycine max | GmSIP2 | C6TE81_SOYBN | 248 | SIP | NPT/NPA | V58 | V181 | P190 | N196 |
| Glycine_max | GmNIP2 | NO26_SOYBN | 271 | NIP | NPA/NPA | W77 | V197 | A206 | R212 |
| Gossypium hirsutum | GohPIP | A0T2N7_GOSHI | 285 | PIP | NPA/NPA | F85 | H214 | T223 | R229 |
| Gossypium hirsutum | GohSIP | D8FSL6_GOSHI | 241 | SIP | NPT/NPA | V52 | V176 | P185 | N191 |
| Haematobia irritans exigua | HiAQPe | AQP_HAEIX | 251 | AQPe | NPA/NPA | F61 | H186 | A195 | R201 |
| Harpegnathos saltator | HasMIP | E2C5P1_9HYME | 278 | unclassified | NPA/NPA | F61 | A193 | C202 | R208 |
| Holophaga foetida | HfAQPp | H1NWW6_9BACT | 229 | AQPp | NPA/NPA | F45 | H174 | T183 | R189 |
| Homo sapiens | HsAQPe | AQP1_HUMAN | 269 | AQPe | NPA/NPA | F56 | H180 | C189 | R195 |
| Hordeum vulgare var distichum | HvTIP | F2EBU5_HORVD | 262 | TIP | NPA/NPA | H66 | I189 | A198 | R204 |
| Juglans_regia | JrPIP | C5IX26_9ROSI | 239 | PIP | NPA/NPA | L83 | H212 | T221 | V227 |
| Lacinutrix sp | LasAQPp | F6GJE1_LACS5 | 229 | AQPp | NPA/NPA | F42 | H172 | T181 | R187 |
| Lactobacillus casei | LcGLAp1 | B3W7H6_LACCB | 235 | GLAp | NPA/NPA | W45 | G174 | Y183 | R189 |
| Lactobacillus casei | LcGLAp2 | Q03AY5_LACC3 | 245 | GLAp | NPA/NPA | Y49 | V181 | P190 | R196 |
| Lactobacillus fermentum | LfGLAp | C0WZ27_LACFE | 240 | GLAp | NPA/NPA | Y49 | V178 | P187 | R193 |
| Lactobacillus plantarum | LpAQPp | C6VMN6_LACPJ | 216 | AQPp | NPA/NPA | F36 | I160 | G169 | R175 |
| Lactobacillus sakei subsp sakei | LsAQPp | Q38UI4_LACSS | 217 | AQPp | NPA/NPA | F36 | I160 | G169 | R175 |
| Lactobacillus_helveticus | LhGLAp | A8YWR0_LACH4 | 250 | GLAp | NPA/NPA | F55 | V187 | P196 | R202 |
| Lotus japonicus | LjNIP | A9YTW6_LOTJA | 302 | NIP | NPS/NPV | A115 | I234 | G243 | R249 |
| Lumbricus rubellus | LrAQPe | C0MP64_LUMRU | 320 | AQPe | NPA/NPA | F95 | H219 | S228 | R234 |
| Marivirga_tractuosa | MatGLAp | E4TR79_MARTH | 243 | GLAp | NPA/NPA | W44 | G182 | Y191 | R197 |
| Medicago truncatula | MtPIP | G7IRS0_MEDTR | 287 | PIP | NPA/NPA | F89 | H218 | T227 | R233 |
| Medicago truncatula | MtSIP1 | B7FN52_MEDTR | 239 | SIP | NPL/NPA | S50 | H170 | G179 | A185 |
| Medicago truncatula | MtSIP2 | G7JDK7_MEDTR | 239 | SIP | NPS/NPA | N52 | V176 | P185 | N191 |
| Medicago truncatula | MtSIP3 | G7KYE4_MEDTR | 246 | SIP | NPV/NPA | V59 | V182 | P191 | N197 |
| Medicago_truncatula | MtNIP | G7KBN5_MEDTR | 268 | NIP | NPA/NPA | W78 | V198 | G207 | R213 |
| Meleagris gallopavo | MgMIP | G1N3T5_MELGA | 187 | putative | NPV/NPA |  | I126 | P135 | R141 |
| Mesorhizobium ciceri | McAQPp | E8TNC7_MESCW | 225 | AQPp | NPA/NPA | F42 | H168 | T177 | R183 |
| Milnesium tardigradum | MtAQPe | G5CTG2_9BILA | 277 | AQPe | NPA/NPA | F60 | H190 | T199 | R205 |
| Mimosa pudica | MpPIP | Q5DVT8_MIMPU | 286 | PIP | NPA/NPA | F88 | H227 | T226 | R232 |
| Mycoplasma conjunctivae | McGLAp | C5J5I0_MYCCR | 247 | GLAp | NPA/NPA | W47 | G181 | Y190 | R196 |
| Myxococcus xanthus | MxAQPp | Q1D2A0_MYXXD | 278 | AQPp | NPA/NPA | F59 | H191 | T200 | R206 |
| •Nannochloropsis gaditana | NgMIP | EKU22600.1* | 230 | - | NPS/NPM | W44 | L162 | P172 | I178 |
| Nematostella vectensis | NvAQPe | A7S514_NEMVE | 248 | AQPe | NPA/NPA | F51 | H175 | C184 | R190 |
| Olea europaea | OeTIP | Q2XVV9_OLEEU | 252 | TIP | NPA/NPA | H65 | I187 | A196 | V202 |
| Opitutus terrae | OtMIP | B1ZTT7_OPITP | 207 | putative | NPA/NPA | I33 | A152 | G161 | V167 |
| Oryza_sativa | OsNIP22 | NP_001057207.1* | 298 | NIP | NPA/NPA | G91 | S210 | G219 | R225 |
| Oryza_sativa | OsNIP21 | NP_001048108.1* | 298 | NIP | NPA/NPA | G88 | S207 | G216 | R220 |
| Paenibacillus polymyxa | PpGLAp | E0RA12_PAEP6 | 274 | GLAp | NPA/NPA | W44 | G173 | Y182 | R188 |
| Pan troglodytes | PatAQPe | H2QUD6_PANTR | 269 | AQPe | NPA/NPA | F56 | H180 | C189 | R195 |
| Parachlamydia acanthamoebae | PaMIP | D1R8W1_9CHLA | 212 | unclassified | NPA/NPA | V40 | A157 | A166 | V172 |
| Patulibacter sp I11 | PsMIP | H0E5C4_9ACTN | 235 | unclassified | NPA/NPA | F59 | H180 | T189 | R195 |
| Pediculus humanus | PhMIP | E0VPZ5_PEDHC | 237 | putative | NPA/NPA | F32 | A162 | C171 | R177 |
| Pediculus humanus subsp corporis | PehAQPe | E0VGZ9_PEDHC | 281 | AQPe | NPA/NPA | F93 | H217 | A226 | R232 |
| Pediococcus claussenii | PcAQPp | G8PEW3_PEDCP | 216 | AQPp | NPA/NPA | F36 | I160 | G169 | R175 |
| Pellia endiviifolia | PeTIP | C9WCK9_9MARC | 254 | TIP | NPA/NPA | H66 | H189 | A198 | R204 |
| Peptostreptococcus anaerobius | PaGLAp | D3MR09_9FIRM | 233 | GLAp | NPA/NPA | W44 | G171 | Y180 | R186 |
| Petunia_hybrida | PhyTIP | Q8W1A5_PETHY | 250 | TIP | NPA/NPA | H65 | I186 | A195 | V201 |
| •Phaeodactylum tricornutum | PtMIP1 | B7FVY0_PHATR | 273 | unclassified | NPA/NPA | W55 | V203 | A212 | R218 |
| •Phaeodactylum tricornutum | PtMIP2 | B7S457_PHATR | 250 | unclassified | NPA/NPV | W42 | V190 | G199 | R205 |
| •Phaeodactylum tricornutum | PtMIP3 | B7G0Y5_PHATR | 270 | unclassified | NPA/NPA | W63 | V210 | A219 | R225 |
| •Phaeodactylum tricornutum | PtMIP4 | B7FRM9_PHATR | 297 | putative | NPG/NPA | F70 | H206 | C215 | R221 |
| •Phaeodactylum tricornutum | PtMIP5 | B7FQU2_PHATR | 291 | putative | NPA/NPM | W72 | L200 | P209 | L215 |
| Photobacterium profundum | PhpMIP | Q1YZY0_PHOPR | 283 | unclassified | NPA/NPA | W50 | G193 | F202 | R208 |
| Physcomitrella patens | PpMIP | A9U3Q2_PHYPA | 279 | putative | NPT/NPA | F49 | T176 | P184 | N190 |
| Physcomitrella patens subsp patens | PpsNIP | A9SLH5_PHYPA | 237 | NIP | NPA/NPA | F48 | A167 | A176 | R182 |
| Physcomitrella patens subsp patens | PpPIP | A9SU06_PHYPA | 280 | PIP | NPA/NPA | F79 | H208 | T217 | R223 |
| Phytophthora infestans | PiGLAe | D0NBP1_PHYIN | 308 | GLAe | NPS/NPA | W102 | G236 | Y245 | R251 |
| Phytophthora sojae | PsGLAe | G5A717_PHYSO | 340 | GLAe | NPA/NPA | W133 | G267 | Y276 | R282 |
| Picea sitchensis | PsPIP1 | A9NPI5_PICSI | 283 | PIP | NPA/NPA | F78 | H207 | T216 | R222 |
| Picea sitchensis | PsTIP | B8LQ31_PICSI | 248 | TIP | NPA/NPA | H62 | I184 | A193 | R199 |
| Picea sitchensis | PsSIP | A9NNF2_PICSI | 238 | SIP | NPT/NPA | F51 | V173 | P182 | N188 |
| Picea sitchensis | PsPIP3 | A9NZ60_PICSI | 275 | PIP | NPA/NPA | F71 | Y200 | T209 | R215 |
| Picea sitchensis | PsNIP | B8LPH5_PICSI | 280 | NIP | NPA/NPA | W83 | V202 | A211 | R222 |
| Picea_abies | PiaPIP | Q3ZDL5_PICAB | 280 | PIP | NPA/NPA | F75 | H204 | T213 | R219 |
| Picea_sitchensis | PsPIP2 | A9P150_PICSI | 265 | PIP | NPA/NPA | F71 | Y200 | T209 | R215 |
| Pisum_sativum | PisNIP | Q9XGG7_PEA | 270 | NIP | NPA/NPA | W78 | V198 | A207 | R213 |
| Plasmodium berghei | PbMIP | Q4YH73_PLABE | 258 | putative | NLA/NPS | W50 | G181 | F190 | R196 |
| Populus trichocarpa | PtNIP1 | B9GUW3_POPTR | 282 | NIP | NPA/NPA | W84 | A207 | A216 | R182 |
| Populus trichocarpa | PtNIP2 | B9H2D3_POPTR | 226 | NIP | NPA/NPA | W39 | V159 | A168 | R174 |
| Populus trichocarpa | PtSIP | B9HDR6_POPTR | 238 | SIP | NPL/NPA | T49 | H168 | G177 | S183 |
| Populus trichocarpa | PotMIP | B9IAG3_POPTR | 241 | unclassified | NPA/NPA | V51 | V177 | P186 | N192 |
| Prevotella melaninogenica | PmAQPp | D9RTS0_PREMB | 235 | AQPp | NPA/NPA | F43 | H173 | T182 | R188 |
| Protochlamydia amoebophila | PamMIP | Q6MD29_PARUW | 232 | unclassified | NPA/NPA | H43 | I169 | A178 | R184 |
| Protopterus annectens | PraGLPe | D0FZC5_PROAN | 295 | GLAe | NPA/NPA | F63 | G203 | Y212 | R218 |
| Providencia rettgeri | PrGLPp | D4C5G0_PRORE | 286 | GLPp | NPA/NPA | W50 | G193 | F202 | R208 |
| Prunus persica | PpsPIP | A5A8K8_PRUPE | 289 | PIP | NPA/NPA | F97 | H226 | T235 | R241 |
| Pseudomonas aeruginosa | PaGLPp | B7VAK1_PSEA8 | 279 | GLPp | NPA/NPA | W51 | G194 | F203 | R209 |
| Pseudomonas putida | PspGLPp1 | B1J4H2_PSEPW | 283 | GLPp | NPA/NPA | W51 | G194 | F203 | R209 |
| Pseudomonas putida | PspGLPp2 | E4R723_PSEPB | 283 | GLPp | NPA/NPA | W51 | G194 | F203 | R209 |
| •Pseudo-nitzschia multiseries | PnmMIP | - | 301 | - | NPA/NPM | W74 | L216 | P225 | L231 |
| Rana nigromaculata | RnAQPe | D0VY82_RANNI | 273 | AQPe | NPA/NPA | F49 | H173 | C182 | R188 |
| Rhizobium_sp | RhMIP | C3KML0_RHISN | 227 | unclassified | NPA/NPA | T49 | A162 | S171 | V177 |
| Ricinus communis | RcPIP | Q70AP4_RICCO | 288 | PIP | NPA/NPA | F96 | H226 | T235 | R241 |
| Ricinus communis | RcTIP1 | B9SI44_RICCO | 255 | TIP | NPA/NPA | H64 | I186 | A195 | R201 |
| Ricinus communis | RcTIP2 | B9RS20_RICCO | 247 | TIP | NPA/NPA | H60 | I181 | A190 | R196 |
| Ricinus communis | RcTIP3 | B9SYP9_RICCO | 252 | TIP | NPA/NPA | H65 | I187 | A196 | V202 |
| Roseibium_sp | RsMIP | E2CJM8_9RHOB | 225 | putative | NPA/NPA | T49 | A162 | S171 | V177 |
| Salinispora arenicola | SaMIP | A8M339_SALAI | 215 | putative | NPA/NPA | I33 | A153 | A162 | V1688 |
| Segniliparus rugosus | SrMIP | E5XPS3_9ACTO | 229 | unclassified | NPA/NPA | I54 | A173 | G182 | V188 |
| Selaginella moellendorffi | SmMIP | D8QX09_SELML | 263 | unclassified | NPA/NPA | H61 | H183 | A192 | R198 |
| Selaginella moellendorffii | SmTIP | D8QSE5_SELML | 244 | TIP | NPA/NPA | H62 | I184 | G193 | R199 |
| Selaginella moellendorffii | SmPIP | D8TFR1_SELML | 284 | PIP | NPA/NPA | F82 | H211 | S220 | R226 |
| Sorghum bicolor | SobNIP1 | C5YGT1_SORBI | 297 | NIP | NPA/NPA | A123 | A245 | P254 | R260 |
| Sorghum bicolor | SobNIP2 | C5XYL0_SORBI | 287 | NIP | NPA/NPA | W87 | V207 | A214 | R221 |
| Sorghum bicolor | SobSIP1 | C5Y892_SORBI | 246 | SIP | NPT/NPA | L57 | I180 | P189 | N195 |
| Sorghum bicolor | SobSIP2 | C5YXN5_SORBI | 243 | SIP | NPT/NPA | L54 | V177 | P186 | N192 |
| Staphylococcus saprophyticus subsp saprophyticus | StsAQPp | Q49UD3_STAS1 | 224 | AQPp | NPA/NPA | F42 | H166 | T175 | R181 |
| Streptococcus gallolyticus | SgGLAp | F5X0W3_STRG1 | 238 | GLAp | NPA/NPA | W44 | G173 | Y182 | R188 |
| Streptococcus sp oral taxon 071 | SsAQPp | E0PZQ5_9STRE | 222 | AQPp | NPA/NPA | F40 | I165 | L174 | R180 |
| Streptomyces_roseosporus | StrAQPp | D6ALV1_STRFL | 237 | AQPp | NPA/NPA | F40 | H170 | T179 | R185 |
| •Synedra acus | SarMIP | G4X445_9STRA | 286 | putative | NPA/NPM | W72 | L198 | P207 | L213 |
| Takifugu rubripes | TrGLAe | H2S528_TAKRU | 297 | GLAe | NPA/NPA | F69 | S209 | A218 | R224 |
| Tetraodon nigroviridis | TnMIP | Q4RVH6_TETNG | 333 | unclassified | NPA/NPA | F69 | H219 | A228 | R234 |
| •Thalassiosira oceanic | ToMIP2 | EJK46256.1* | 330 | - | NPA/NPA | I138 | A257 | G266 | V272 |
| •Thalassiosira oceanica | ToMIP1 | EJK66519.1* | 283 | - | NPS/NPM | W69 | L196 | P205 | L211 |
| •Thalassiosira pseudonana | TpMIP1 | B8BSU4_THAPS | 219 | unclassified | NPA/NPA | I33 | A152 | G161 | V167 |
| •Thalassiosira pseudonana | TpMIP2 | B8BU47_THAPS | 280 | putative | NPA/NPM | W70 | I197 | P206 | L212 |
| Thiocapsa_marina | TmAQPp | F9UH73_9GAMM | 245 | AQPp | NPA/NPA | F45 | H183 | T192 | R198 |
| Tolumonas auensis | TaMIP | C4LFY0_TOLAT | 280 | unclassified | NPA/NPA | W48 | G191 | F200 | R206 |
| Tribolium castaneum | TcaAQPe | D6X3I5_TRICA | 248 | AQPe | NPA/NPA | F61 | H185 | S194 | R200 |
| Trypanosoma cruzi | TcAQPe | Q4DX44_TRYCR | 231 | AQPe | NPA/NPA | I39 | S159 | G168 | V174 |
| uncultured Bacteroidetes bacterium 'SBI2-18 P41A3' | SbGLAp | Q2VBU6_9BACT | 244 | GLAp | NPA/NPA | W45 | G184 | F193 | R199 |
| Uncultured methanogenic archaeon RC-I | ArMIP | Q0W089_UNCMA | 263 | putative | NPA/NPA | F62 | I186 | S195 | R201 |
| Vibrio mimicus | VmGLPp | D2Y9L0_VIBMI | 284 | GLPp | NPA/NPA | W50 | G193 | F202 | R208 |
| Vibrio sp EJY3 | VeGLPp | H2IEK6_9VIBR | 273 | GLPp | NPA/NPA | W46 | G189 | F198 | R204 |
| Vitis vinifera | VvNIP | D7TJR3_VITVI | 282 | NIP | NPA/NPA | W86 | V206 | A215 | R221 |
| Vitis vinifera | VvPIP | Q0MX12_VITVI | 287 | PIP | NPA/NPA | F87 | H216 | T225 | R231 |
| Vitis vinifera | VvTIP | D7T7C6_VITVI | 259 | TIP | NPA/NPA | H66 | I188 | A197 | R203 |
| Vitis_vinifera | VvSIP | A5C3B1_VITVI | 221 | SIP | NPI/NPA | I36 | V159 | P168 | N174 |
| Weissella confusa | WcGLAp | H1X9V3_LACCO | 239 | GLAp | NPA/NPA | Y49 | V177 | P186 | R192 |
| Xanthomonas vesicatoria | XvGLPp | F0BFN5_9XANT | 273 | GLPp | NPA/NPA | W49 | G190 | W199 | R206 |
| Xenopus laevis | XlMIP | Q5EAY3_XENLA | 297 | unclassified | NPA/NPA | F63W | G203 | Y212 | R218 |
| Xenopus tropicalis | XtMIP | F6QCC5_XENTR | 284 | unclassified | NPA/NPA | F63 | G194 | Y203 | R209 |
| Xenopus tropicalis | XtAQPe | F6RVG6_XENTR | 267 | AQPe | NPA/NPA | F48 | H172 | C181 | R187 |
| Yersinia enterocolitica | YeGLPp | F4N0I8_YEREN | 199 | GLPp | NPA | W46 | G189 | L198 |  |
| Yersinia_kristensenii | YkAQPp | C4TYD1_YERKR | 234 | AQPp | NPA/NPA | F42 | H172 | T181 | R187 |
| Zea mays | ZmNIP | B6SL51_MAIZE | 277 | NIP | NPA/NPI | V71 | G188 | G197 | R203 |
| Zea mays | ZmNIP22 | NP_001105020.1* | 294 | NIP | NPA/NPA | G91 | S210 | G219 | R225 |
| Zea mays | ZmNIP23 | NP_001105517.1* | 301 | NIP | NPA/NPA | G94 | S213 | G222 | R228 |
| Zea mays | ZmNIP21 | NIP21_MAIZE | 295 | NIP | NPA/NPA | G86 | S205 | G214 | R222 |
| Zea mays | ZmTIP | TIP32_MAIZE | 266 | TIP | NPA/NPA | H74 | V196 | A205 | R211 |
| Zea mays | ZmSIP1 | B6TIZ3_MAIZE | 243 | SIP | NPT/NPA | L54 | V177 | P186 | N192 |
| Zea mays | ZmSIP2 | SIP11_MAIZE | 245 | SIP | NPT/NPA | L56 | I179 | P188 | N194 |

* - accession number of GenBank
